# In-Depth Specificity Profiling of Endopeptidases Using Dedicated Mix-and-Split Synthetic Peptide Libraries and Mass Spectrometry

**DOI:** 10.1021/acs.analchem.3c01215

**Published:** 2023-07-26

**Authors:** Bart Claushuis, Robert A. Cordfunke, Arnoud H. de Ru, Annemarie Otte, Hans C. van Leeuwen, Oleg I. Klychnikov, Peter A. van Veelen, Jeroen Corver, Jan W. Drijfhout, Paul J. Hensbergen

**Affiliations:** † Center for Proteomics and Metabolomics, Leiden University Medical Center, Leiden, 2333 ZA, The Netherlands; ‡ Department of Immunology, Leiden University Medical Center, Leiden, 2333 ZA, The Netherlands; § Department of CBRN Protection, Netherlands Organization for Applied Scientific Research TNO, Rijswijk, 2280 AA, The Netherlands; ∥ Department of Biochemistry, Moscow State University, Moscow 119991, Russian Federation; ⊥ Department of Medical Microbiology, Leiden University Medical Center, Leiden, 2333 ZA, The Netherlands

## Abstract

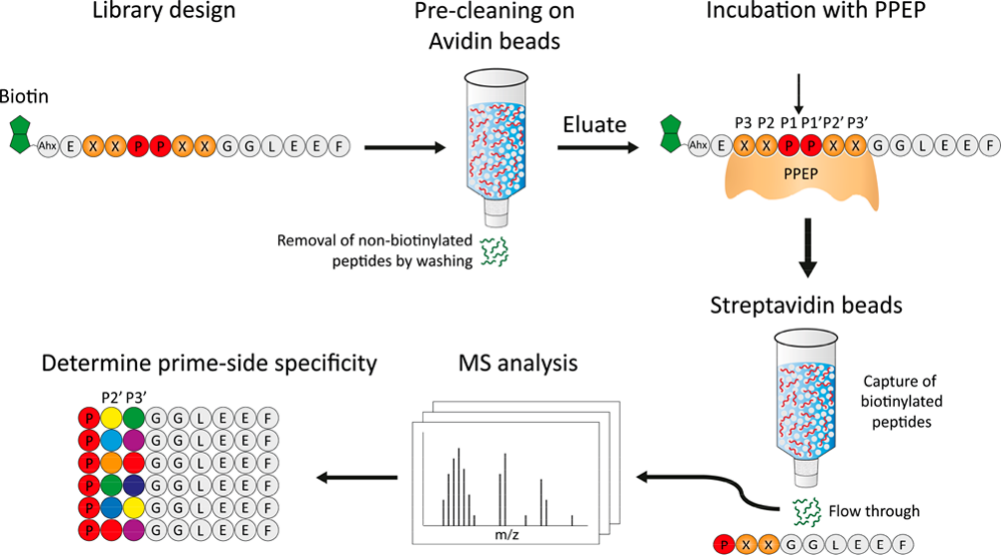

Proteases comprise the class of enzymes that catalyzes the hydrolysis of peptide bonds, thereby playing a pivotal role in many aspects of life. The amino acids surrounding the scissile bond determine the susceptibility toward protease-mediated hydrolysis. A detailed understanding of the cleavage specificity of a protease can lead to the identification of its endogenous substrates, while it is also essential for the design of inhibitors. Although many methods for protease activity and specificity profiling exist, none of these combine the advantages of combinatorial synthetic libraries, i.e., high diversity, equimolar concentration, custom design regarding peptide length, and randomization, with the sensitivity and detection power of mass spectrometry. Here, we developed such a method and applied it to study a group of bacterial metalloproteases that have the unique specificity to cleave between two prolines, i.e., Pro-Pro endopeptidases (PPEPs). We not only confirmed the prime-side specificity of PPEP-1 and PPEP-2, but also revealed some new unexpected peptide substrates. Moreover, we have characterized a new PPEP (PPEP-3) that has a prime-side specificity that is very different from that of the other two PPEPs. Importantly, the approach that we present in this study is generic and can be extended to investigate the specificity of other proteases.

## Introduction

Proteases comprise a class of enzymes that catalyzes the hydrolysis of peptide bonds between amino acids in a polypeptide chain. Through cleavage of their substrates, proteases play a pivotal role in many aspects of life, ranging from viral polyprotein processing^1^ to a wide range of human physiological and cellular processes, e.g., hemostasis, apoptosis, and immune responses.^2−4^ Uncovering the endogenous substrate(s) is usually a key step toward dissecting the biological role of a protease. However, it is not straightforward to identify protease substrates without prior knowledge, e.g., without a clear phenotype in a protease knockout or lack of information from homologues in other species. Information about the cleavage specificity of a protease can aid in the identification of endogenous substrates. Moreover, such information is pivotal for inhibitor design or the development of diagnostic biomarker assays.^5−7^

**Figure 1:**
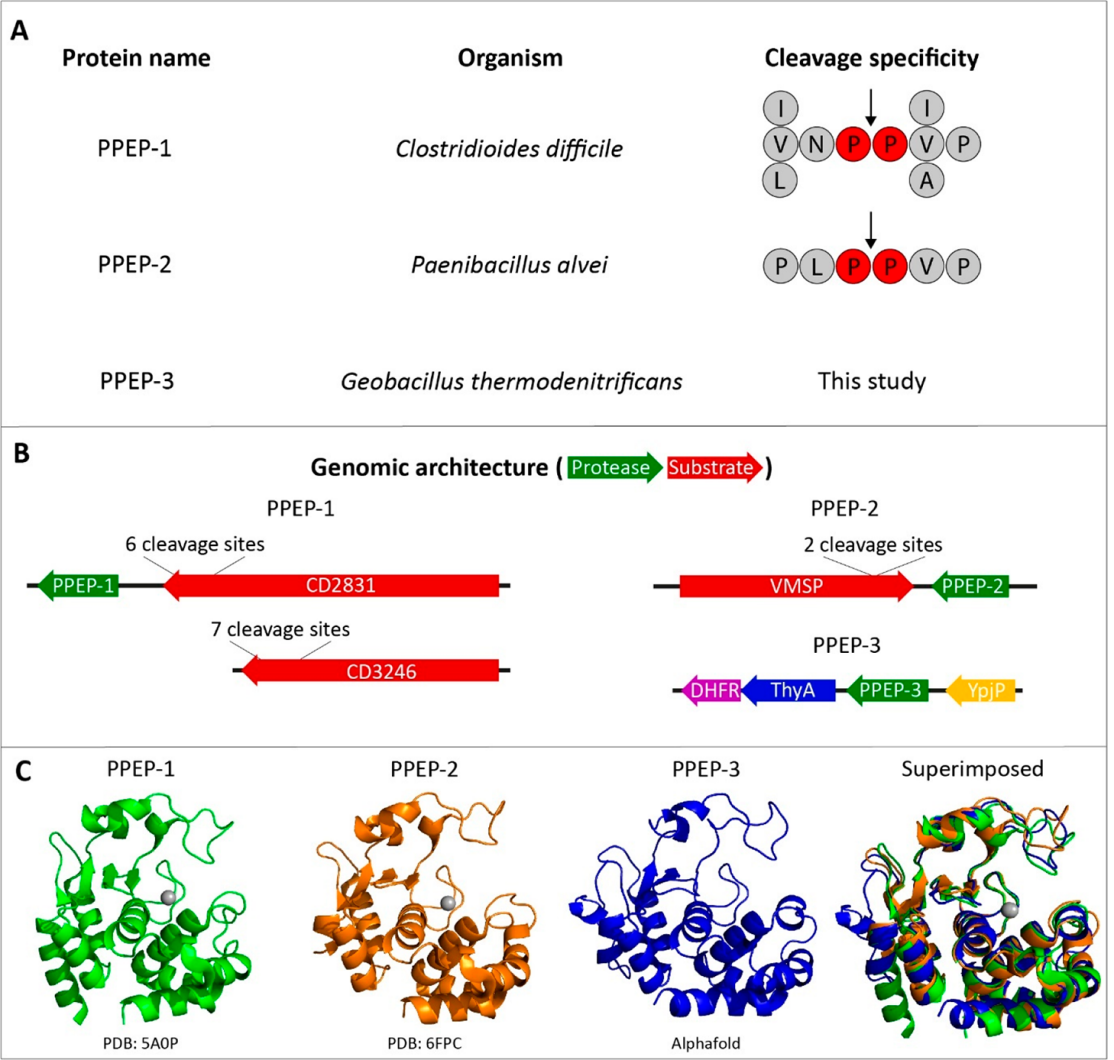
Overview of the PPEPs used in this study. (A) The three PPEPs that are used in this study and their respective origins and substrate specificity. For PPEP-1 and 2 the cleavage specificity is based on the endogenous substrates. For PPEP-3, no substrates have been described yet. (B) The genomic architecture of the PPEPs and their substrates. For PPEP-1, the gene encoding the substrate CD2831 is adjacent to PPEP-1. The gene encoding the second substrate (CD3246) is positioned elsewhere in the genome. The genes for PPEP-2 and its substrate VMSP are located adjacent to each other. For PPEP-3, no adjacent genes contain the consensus PPEP cleavage motif (i.e., PPXP). (C) Crystal (PPEP-1 and PPEP-2) and predicted (PPEP-3) structures.^9,10^ PPEP-3 structure was predicted using the Alphafold algorithm.^35^

We study a group of bacterial proteases that have the unique specificity to cleave a peptide bond between two prolines, i.e., Pro-Pro endopeptidases (PPEPs). The first two members, PPEP-1 from the human pathogen *Clostridioides difficile*(8,9) and PPEP-2 from *Paenibacillus alvei*,^10^ are secreted enzymes that cleave cell surface proteins involved in bacterial adhesion. Initially, the specificity of PPEP-1 was determined based on a small synthetic peptide library that was designed based on the identification of a suboptimal cleavage site in a human protein.^11^ Following the elucidation of the endogenous PPEP-1 substrates, in which a total of 13 cleavage sites were found, a cleavage motif was determined (Figure 1A). For PPEP-2, the endogenous cleavage site (Figure 1A) was experimentally determined following an *in silico* prediction of the substrate. This prediction was based on a similar genomic organization of the PPEP gene and its substrate in both *C. difficile* and *P. alvei*, i.e., they are adjacent genes (Figure 1B). Based on a bioinformatic analysis, we recently observed PPEP homologues in a wide variety of species,^12^ for example, in *Geobacillus thermodenitrificans* (PPEP-3, Figure 1). The modeled structure of PPEP-3 shows a high degree of similarity to the crystal structures of PPEP-1 and PPEP-2 (Figure 1C). However, none of the genes adjacent to PPEP-3 encode a protein that contains a PPEP consensus cleavage motif (XXPPXP, Figure 1A,B), hampering the formulation of a testable hypothesis about its substrate(s). Hence, to gain insight in the activity and specificity of hitherto uncharacterized putative PPEPs, a general method to profile their specificity is needed.

A wide variety of methods for protease activity and specificity profiling has been developed.^6,13,14^ Several strategies rely on the identification of protease-generated protein neo-*N*-termini in cells expressing the protease of interest compared to controls. For this purpose, positive and negative selection procedures for the enrichment of *N*-terminal peptides in combination with quantitative mass spectrometry based proteomics methods, collectively known as *N*-terminomics, have been developed.^15−20^ However, for an optimal experimental setup for such an experiment, a protease knockout cell line or strain is necessary.

Other strategies seek to identify the protease substrate specificity by making use of peptide libraries, either by phage display technologies^21,22^ or as a collection of (synthetic) peptides. For the latter, mass spectrometry analysis is an attractive readout because it determines the signature proteolytic event in a highly specific manner, i.e., information on the amino acid(s) surrounding the scissile bond is obtained. For example, MALDI-based approaches using synthetic peptide arrays have been used to profile protease activity and specificity, but for such approaches, each peptide requires individual synthesis, treatment, and analysis.^23,24^ In addition, proteome-derived peptide libraries have been shown to be a rich source of peptides for these types of analyses.^25−27^ Although with this method a wide variety of potential substrates is tested in a single reaction, the concentration range of the peptides present may easily span a few orders of magnitude. This may complicate the assessment of whether a product peptide is derived from a very good substrate present at a low concentration or a poor substrate at a high concentration instead. Another method, MSP-MS, uses a small set of synthetic peptides in which amino acid pairs are cleverly positioned in order to contain a wide variety of potential cleavage sites.^28^ However, this design was based on the assumption that, for a protease, only the correct positioning of two amino acids is necessary for a protease to cleave its substrate. Based on the inspection of the list of 228 peptides,^29^ we predict that none of these would be cleaved by one of the PPEPs, making MSP-MS not suitable for specificity profiling of PPEPs and probably other proteases as well.

The combination of equimolar peptide concentrations with high diversity would be the ideal scenario for the design of a peptide library. This can be achieved by constructing a synthetic combinatorial peptide library, for example using the one-bead-one-compound approach,^30^ and such libraries have been used to profile protease specificity.^31,32^ As a read-out for the cleavage of peptides, both fluorescence detection^5,31^ and Edman degradation^33,34^ have so far been used.

Given the beneficial characteristics of mass spectrometry mentioned above, we reasoned that it would be highly advantageous if this could be applied to analyze the product peptides following the incubation of a combinatorial synthetic peptide library with a protease of interest in a single reaction, but this has hitherto not been done. Obviously, the complexity of combinatorial libraries tends to increase dramatically when multiple positions are randomized, thereby impeding MS analysis. Therefore, two aspects are pivotal to make such an approach suitable. First of all, in the design of the library, any prior knowledge or hypothesis about the protease specificity should be utilized. Second, a strategy to enrich, analyze, and identify the product peptides has to be implemented.

Therefore, the aim of the current study was to develop a novel method to study the activity and specificity of a protease, which combines the advantages of a combinatorial synthetic peptide library, i.e., high diversity and equimolar peptide concentrations, with the sensitivity and specificity of MS detection. Testing the method with PPEP-1 and PPEP-2 showed results that were in good agreement with previous data, while some unexpected peptide substrates were observed. Importantly, the new method clearly established PPEP-3 as a genuine PPEP, but also showed that it has a markedly different prime-side specificity compared to that of PPEP-1 and PPEP-2.

## Experimental Section

### Combinatorial Peptide Library Assays

For details on the synthesis of the combinatorial peptide library, see Supporting Information. To remove nonbiotinylated peptides, 50 nmol of peptides from the (sub)library (5 μL 10 nmol/μL stock in 1 mL of PBS) was loaded onto a 3 mL filter column containing 1 mL of Pierce Monomeric Avidin Agarose beads (Thermo; binding capacity is >1.2 mg/mL biotinylated BSA or >18 nmol/mL). Prior to loading the libraries, the avidin column was washed five times with 1 mL of 0.1 M glycine (pH 2.7) and subsequently washed five times with 1 mL of PBS. After loading peptides, the flow-through was collected. Next, 1 mL of PBS was loaded onto the column and flow-through was collected. Then, the collected flow-throughs were reapplied to the column to ensure saturation of the avidin beads. The column was washed five times with 1 mL of PBS to remove nonbiotinylated peptides. Next, 1 mL of 0.1 M glycine (pH 2.7) was applied to the column and the flow-through was discarded because the pH of the last drop of this fraction was still neutral as checked with a pH indicator strip. Then, biotinylated peptides were eluted with 9 mL of 0.1 M glycine (pH 2.7). Eluted peptides were desalted using reversed-phase solid phase extraction cartridges (Oasis HLB 1 cm^3^ 30 mg, Waters) and eluted with 400 μL of 50% acetonitrile (v/v) in 0.1% formic acid. Samples were dried by vacuum concentration and stored at −20 °C until further use. If the binding efficiency of the avidin beads is the same for the peptide library as for biotinylated BSA, and no peptides are lost during the prewash steps, we expect approximately 20 nmol of peptide yield after the avidin preclearing step.

Precleaned (sub)libraries (approximately 10 nmol) were incubated with a PPEP (200 ng) for 3 h at 37 °C in PBS. A nontreated control was included. After incubation, the samples were loaded onto an in-house constructed column consisting of a 200 μL pipet tip containing a filter and a packed column of 100 μL of Pierce High Capacity Streptavidin Agarose beads (Thermo, column was washed four times with 150 μL of PBS prior to use), in order to remove the biotinylated peptides. The flow-through and four additional washes with 125 μL were collected. The resulting product peptides were desalted using reversed-phase solid phase extraction cartridges (Oasis HLB 1 cm^3^ 30 mg, Waters) and eluted with 400 μL of 30% acetonitrile (v/v) in 0.1% formic acid. Samples were dried by vacuum concentration and stored at −20 °C until further use.

### LC-MS/MS Analyses

Product peptides were analyzed by online C18 nanoHPLC MS/MS with a system consisting of an Ultimate3000nano gradient HPLC system (Thermo, Bremen, Germany), and an Exploris480 mass spectrometer (Thermo). Fractions were injected onto a cartridge precolumn (300 μm × 5 mm, C18 PepMap, 5 μm, 100 A, and eluted via a homemade analytical nano-HPLC column (50 cm × 75 μm; Reprosil-Pur C18-AQ 1.9 μm, 120 A; Dr. Maisch, Ammerbuch, Germany). The gradient was run from 2% to 36% solvent B (20/80/0.1 water/acetonitrile/formic acid (FA) v/v) in 52 min. The nano-HPLC column was drawn to a tip of ∼10 μm and acted as the electrospray needle of the MS source. The mass spectrometer was operated in data-dependent MS/MS mode for a cycle time of 3 s, with a HCD collision energy at 30 V and recording of the MS2 spectrum in the orbitrap, with a quadrupole isolation width of 1.2 Da. In the master scan (MS1) the resolution was 120000, the scan range 350–1600, at standard AGC target at maximum fill time of 50 ms. A lock mass correction on the background ion *m*/*z* = 445.12003 was used. Precursors were dynamically excluded after n = 1 with an exclusion duration of 10 s and with a precursor range of 10 ppm. Charge states 1–5 were included. For MS2 the first mass was set to 110 Da, and the MS2 scan resolution was 30,000 at an AGC target of 100%@maximum fill time of 60 ms.

### LC-MS/MS Data Analysis

We generated a database containing all 6859 peptides from the P3 = Val sublibrary, i.e., Ahx-EVXPPXXGGLEEF. The Ahx in all peptide sequences was replaced by a Ile (they have an identical mass). Raw data were converted to peak lists using Proteome Discoverer version 2.4.0.305 (Thermo Electron) and submitted to the in-house created P3 = Val sublibrary database using Mascot v. 2.2.7 (www.matrixscience.com) for peptide identification, using the Fixed Value PSM Validator. Mascot searches were with 5 ppm and 0.02 Da deviation for precursor and fragment mass, respectively, and no enzyme specificity was selected. Biotin on the protein N-terminus was set as a variable modification. Raw data analysis was performed in the Xcalibur Qual Browser (Thermo). The EICs displaying all PXPGGLEEF/PPXGGLEEF peptides were created by plotting the intensities of the signal corresponding to the monoisotopic *m*/*z* values of both 1+ and 2+ charged peptides. To assign individual peptides to their respective peaks, each individual peptide was plotted in an EIC and peptides were assigned to peaks based on retention time and abundance.

## Results and Discussion

### Combinatorial Peptide Library Design and Experimental Setup

**Figure 2:**
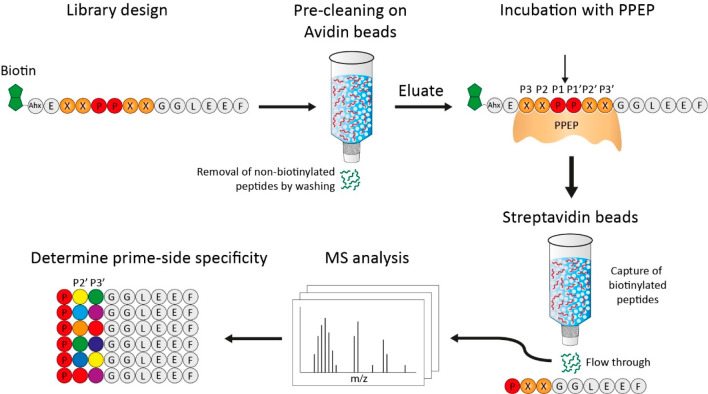
Design of the synthetic combinatorial peptide library and workflow to determine the activity and prime-side specificity of a Pro-Pro endopeptidase (PPEP). The library was designed to contain an XXPPXX motif, with X representing any residue (X ≠ Cys). At the N-terminus, peptides were modified with a biotin, allowing removal of uncleaved peptides and N-terminal product peptides after incubation of the library with a protease, i.e., PPEP. At the C-terminus, a peptide tail (GGLEEF) was added in order for the C-terminal cleavage products to be compatible with LC-MS/MS analysis. This stretch of amino acids was also chosen based on a previously recorded MS/MS spectrum, showing favorable fragmentation characteristics (Figure S1). First, the library was precleaned on avidin beads to remove nonbiotinylated peptides. Then, the library was incubated with a PPEP. The scissile bond is indicated by the arrow. Following this, biotinylated peptides (noncleaved peptides and N-terminal product peptides) were captured on a streptavidin column. The flow-through, containing nonbiotinylated C-terminal product peptides (PXXGGLEEF) were then analyzed by LC-MS/MS, after which the prime-side specificity could be determined. Ahx: 1-aminohexanoic acid.

Since PPEPs are defined by their ability to hydrolyze Pro-Pro bonds, and substrate specificity is further determined by positions P3–P3′ surrounding the scissile bond,^9,11,36^ we constructed a combinatorial peptide library containing a XXPPXX motif. In this motif, the X positions represent any amino acid residue (with the exception of cysteine), while the core proline (P) residues (corresponding to the P1–P1′ positions) are fixed (Figure 2).

In order to analyze product peptides after incubation of the library with a PPEP, the core sequence (XXPPXX) was modified in two ways. First, a six amino acid tail consisting of Gly-Gly-Leu-Glu-Glu-Phe (GGLEEF) was added at the C-terminus (Figure 2). This sequence was chosen because PPEP cleavage between the two prolines would then provide retention of the C-terminal product peptides (PXXGGLEEF) on a C18-column. Moreover, the fragmentation pattern of such a peptide (PYVGGLEEF) that we observed in a previous study provided good sequence coverage of the N-terminal region (Figure S1). Second, a biotin was attached to the N-terminus of each peptide, connected to the rest of the peptide by a small linker (Ahx-Glu, Ahx = 1-aminohexanoic acid, Figure 2). This allows for the enrichment of C-terminal product peptides by removal of biotinylated peptide molecules, i.e., noncleaved peptides and N-terminal product peptides, using streptavidin beads. This is similar to a previous approach which used Edman degradation instead of mass spectrometry to sequence the protease generated product peptides.^37^ In addition to the lower sensitivity of this method, several amino acids could not be accurately detected and information on subsite cooperativity^38^ is lost.

Synthesis of the library was performed using the one-bead one-compound (OBOC) method^30^ in order to achieve equimolar amounts of each unique peptide. Initially, we synthesized 19 sublibraries for which the amino acid at the X corresponding to the P3 position (the first X in the sequence XXPPXX) was known. Each of these sublibraries contains 6859 peptides (19 × 19 × 19). Since the process of linking biotin to the N-terminus is not 100% efficient, nonbiotinylated peptides were also present. To remove these unwanted peptides prior to incubation with a PPEP, the library was precleaned on an avidin column (Figure 2). The biotinylated peptide library that was obtained after elution from the avidin column was then incubated with PPEP and subsequently depleted for biotinylated peptides using streptavidin. C-terminal, nonbiotinylated, product peptides (PXXGGLEEF) were collected in the flow-through and analyzed by mass spectrometry. Peptide identification was accomplished using standard database searching (see Experimental Section for details). Following this, the amino acids at the P2′ and P3′ positions were determined (Figure 2).

### Incubation of PPEP-1 with Two Sublibraries Confirms the Preference of PPEP-1 for Valine over Lysine at the P3 Position

**Figure 3:**
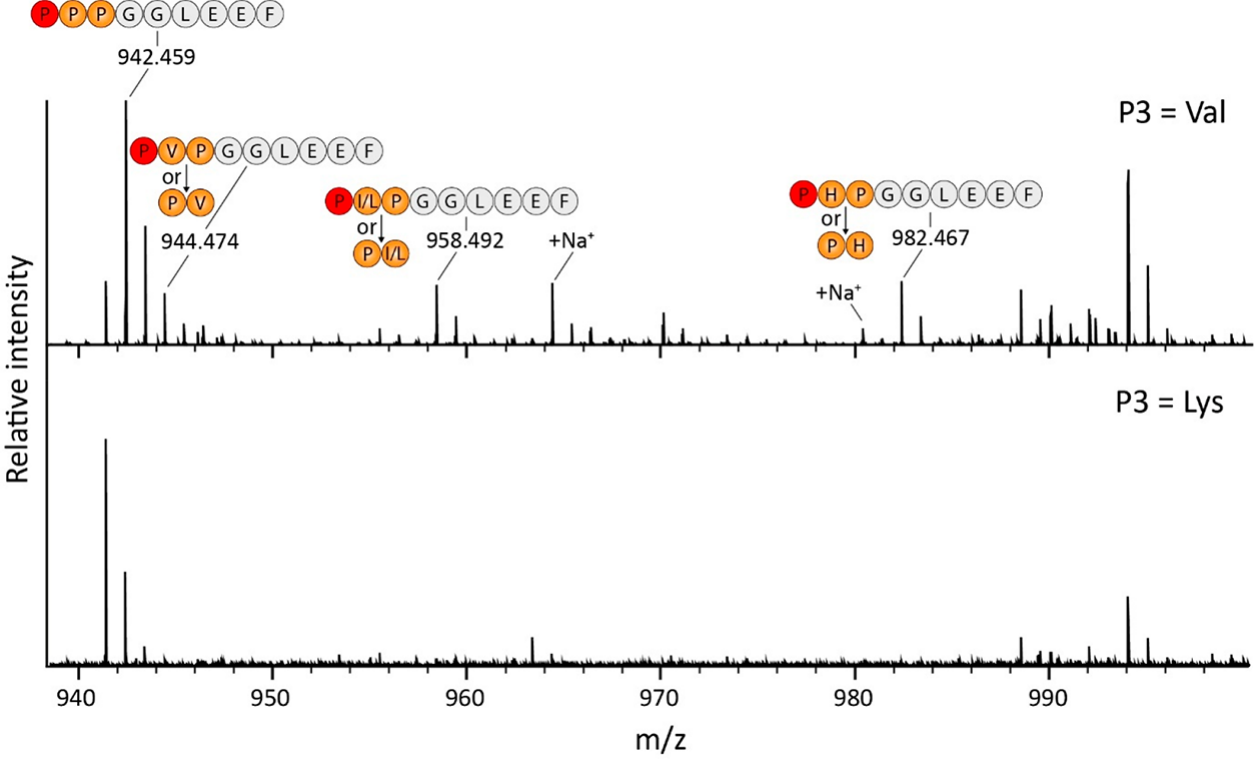
MALDI-FT-ICR MS analysis of PPEP-1 product peptides using two different combinatorial sublibraries. The P3 = Val and P3 = Lys sublibraries were incubated with PPEP-1 for 3 h. Following depletion of biotinylated peptides, nonbiotinylated product peptides (PXXGGLEEF) were analyzed using MALDI-FT-ICR MS. The two indicated sodiated species are from the PPPGGLEEG and P(I/L)PGGLEEF/(PP(I/L)GGLEEF peptides, respectively.

In our previous studies, we showed a preference of PPEP-1 for a Val as compared to a Lys at the P3-position.^36^ Hence, to test the feasibility of our approach, two sublibraries with either a Val or Lys at this position were incubated with PPEP-1. The formation of products due to proteolysis of substrate peptides present in the library was assessed by using MALDI-FT-ICR MS (Figure 3**)**. As expected, product peptides were clearly visible when using the P3 = Val library (Figure 3, top panel), while these were not observed when the P3 = Lys library was used instead (Figure 3, lower panel).

Although no fragmentation was performed, we could assign several product peptides when using the P3 = Val library based on the accurate mass and our current understanding of the specificity of PPEP-1 (Figure 1),^11,36^ i.e., we were expecting PXPGGLEEF peptides. The highest signal was observed for the PPPGGLEEF peptide (*m*/*z* = 942.459, [M + H]^+^). Although three prolines at P1′-P3′ are not found in the endogenous substrates (Figure 1), it had been demonstrated that PPEP-1 prefers all prolines at these positions.^11^ In addition, a peptide matching with the product peptide PIPGGLEEF was observed, although based on the MALDI-FT-ICR MS analysis alone we cannot exclude the possibility that it corresponds to PPIGGLEEF, nor that it might contain a leucine instead of an isoleucine at the site corresponding to the P2′/P3′ position. We also observed a peptide corresponding to PVPGGLEEF (or PPVGGLEEF). Even though the signal for this peptide partially overlapped with the second isotope peak of the PPPGGLEEF peptide (theoretical *m*/*z* value: 944.462, [M + H]^+^), a separate peak for the signal at *m*/*z* 944.474 ([M + H]^+^) was clearly visible. Lastly, a peptide was observed corresponding to either PHPGGLEEF or PPHGGLEEF even though it was hitherto unknown that PPEP-1 allows for a histidine at the P2′ or P3′ position.

Overall, the above results with the two combinatorial sublibraries demonstrated the applicability of our approach to detect PPEP activity and study its preference for amino acids surrounding the scissile Pro-Pro bond.

### PPEP-1, PPEP-2, and PPEP-3 Display Distinct Substrate Specificity after Incubation with the Full Combinatorial Peptide Library

Following the successful tests of the method with the two sublibraries and PPEP-1, we applied our method with the full combinatorial peptide library (a mix of all 19 sublibraries, containing 130321 peptides) to determine the prime-side substrate specificity of PPEP-1, PPEP-2, and PPEP-3. In order to increase the sensitivity and include fragmentation of the product peptides, samples were analyzed with LC-MS/MS. A nontreated sample was included as a control.

Initially, we analyzed the results by standard database searching against an in-house-generated database (see Experimental Section for details). For PPEP-1 and PPEP-2 treated samples, the peptides with the highest intensities represented the expected PXXGGLEEF product peptides (Table S1). Moreover, an enrichment for prolines at the P2′ and/or P3′ positions was observed (Table S1), in line with what was expected based on the specificity of PPEP-1 and PPEP-2 (Figure 1). For the PPEP-3 treated sample, the most highly abundant peptide was PPPGGLEEF. Hence, this clearly demonstrated that also PPEP-3 is an authentic PPEP. In addition, other 9-mer PXXGGLEEF product peptides were present among the most abundant peptides in the PPEP-3 treated sample (Table S1).

The results from the database search showed ambiguity in the position of the proline at the P2′/P3′ position as assigned by the search algorithm (i.e., PXPGGLEEF or PPXGGLEEF). Also, several MS/MS spectra were matched with sequences that did not match the expected 9-mer PXXGGLEEF sequence. For example, some MS/MS spectra were assigned to the 8-mer sequence KYGGLEEF. However, we argue that these represent wrong annotations due to the fact that the mass and elution time of this peptide is exactly the same as the PPPGGLEEF peptide, (one of) the highest product peptides observed for all three PPEPs (Table S1). Furthermore, in all cases where an isoleucine or leucine was present at the P2′ or P3′ position, obviously no distinction could be made by the search algorithm.

To substantiate our results, we combined a manual inspection of the MS/MS spectra with additional LC-MS/MS analyses of a set of synthetic peptides. First of all, KYGGLEEF/YKGGLEEF peptides elute much earlier than the PPPGGLEEF peptide, and the fragmentation of such peptides is very distinct from PXXGGLEEF peptides, PPPGGLEEF in particular (Figure S2). Second, fragmentation spectra of PXPGGLEEF and PPXGGLEEF peptides showed clear differences (Figure S3). Importantly, the spectra of PXPGGLEEF peptides are dominated by the unique PGGLEEF (y_7_) fragment ion (*m*/*z* 748.351, Figure S3). This was, for example, essential in distinguishing PIPGGLEEF from PPIGGLEEF. The other unique fragment ion of PXPGGLEEF peptides, i.e., the b_2_ corresponding to PX, appeared less informative because it could also represent nondiscriminatory internal fragments. We believe that this was one of the reasons why the results from the database searches were often ambiguous. Possibly other search algorithms, or training thereof, and new developments for prediction of tandem MS spectra^39^ could aid in the correct assignment of product peptides in terms of the amino acids at the second and third position in the protease-generated product peptides.

In addition to peptide fragmentation characteristics, separation of isomeric peptides using our reversed-phase chromatography system as part of the LC-MS/MS system was also essential. For example, we observed that peptides with an isoleucine elute earlier than the isomeric peptide having a leucine (Figure S4B,C), in line with what is known about the relative contribution of these two residues to the retention on a reversed phase column.^40^ Another way to discriminate between these two options is by using a stable isotope labeled leucine/isoleucine during the synthesis of the library. PXPGGLEEF and PPXGGLEEF peptide pairs with an identical X residue that we have tested were well separated, with the exception of PIP and PPI (Figure S4). For example, histidine containing peptides were separated depending on the position of the histidine within the peptide, as also observed previously.^41^

**Figure 4:**
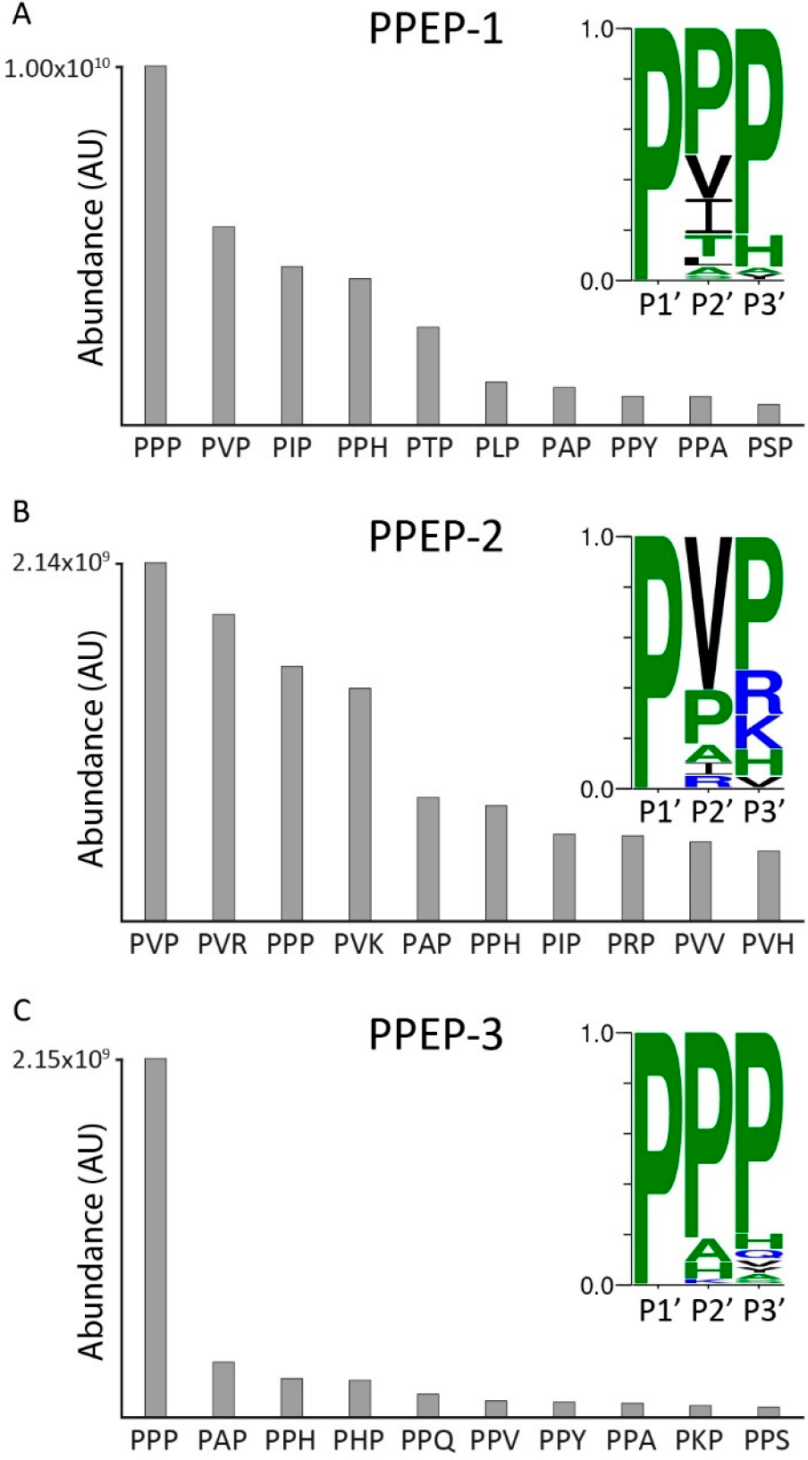
Top 10 most highly abundant 9-mer product peptides of PPEP-1, -2, and -3 reveal differences in prime-side specificity. The full combinatorial peptide library was incubated with recombinant PPEP-1, PPEP-2, or PPEP-3. Product peptides were analyzed using LC-MS/MS. Abundances were determined by summing the intensities of singly and doubly charged peptides. Discrimination between PXP and PPX peptides relied on both inspection of fragmentation spectra and C18 column separation (Figures S3 and S4). The 10 most highly abundant 9-mer product peptides formed by PPEP-1 (A), PPEP-2 (B), and PPEP-3 (C) and their abundances are represented as bars. A cleavage motif was constructed based on the relative intensities of the products peptides. The sequence on the *X*-axis represents the P1′-P3′ residues of the PXXGGLEEF product peptides.

Based on these additional analyses, we could refine the results from the database search and accurately assign the identity and abundance of the individual product peptides. Because, as opposed to proteome-derived peptide libraries,^42,43^ peptides in our library are present in equimolar concentrations, the relative abundance of the individual product peptides enabled us to obtain an estimate of how well specific amino acids are tolerated at the prime sites (Figure 4). However, the difference in intensities between the signals of the individual product peptides in the MS data also relate to how well these peptides are ionized, especially when extra basic amino acids are present, i.e., histidine, arginine, and lysine.^44^ We believe that this could explain the relatively high contribution of these amino acids to the prime-side cleavage motifs that we have obtained (Figure 4). Because most of the total intensity of the 9-mer product peptides could be explained by the 10 most abundant ones, we focused on these. Of note, since the proline at the P1′ was fixed (Figure 2), no variation is observed at this position in Figure 4. We also observed longer peptides (Table S1) but given the large number of isomeric peptides and the extra efforts needed to correctly assign the amino acid sequence for the PXXGGLEEF peptides as described above, we decided to not include these in the further analysis of the prime-side specificity. Notwithstanding, they could potentially also provide some information about the P1′ specificity when looking at the 11-mer peptides.

The prime-side residues of the endogenous substrates of PPEP-1 (Figure 1) were all represented among the top 10 product peptides, again demonstrating the feasibility of our method. In addition, the preference of PPEP-1 to hydrolyze substrates with three prolines at the P1′-P3′ (Figure 3)^11^ was also demonstrated using the full combinatorial library (Figure 4A). Interestingly, our approach revealed several previously unknown prime-side options that allow for cleavage by PPEP-1. The most striking findings included the cleavage of substrates that had either PPH, PPA, or PPY at their P1′-P3′ positions (Figure 4A), since the presence of a Pro residue at P3′ was thought to be a determinant for proteolytic activity.^9,11^ The requirement for a Pro residue at P3′ was explained by the presence of a diverting loop in the cocrystal structure of PPEP-1 with a substrate peptide.^9^ The Pro at P3′ aligns with Trp-103 of PPEP-1 due to a parallel aliphatic-aromatic interaction, thereby redirecting the remainder of the substrate (P4′ and onward) out of the binding pocket by inducing a kink at the P2′ position. Therefore, it was initially hypothesized that the PHPGGLEEF/PPHGGLEEF product observed using MALDI-FT-ICR MS (Figure 3) would in fact be PHPGGLEEF. However, manual inspection of the MS/MS fragmentation spectra revealed that PPEP-1 does tolerate PPH but not PHP at the P1′-P3′ sites. To corroborate this finding, we synthesized two FRET-quenched peptides (Lys_Dabcyl_-EVNPPHPD-Glu_Edans_ and Lys_Dabcyl_-EVNPPPHD-Glu_Edans_) and tested them with PPEP-1. As expected, based on our library results, PPEP-1 is able to hydrolyze a VNP↓PPH, but not a VNP↓PHP peptide (Figure S5). Notwithstanding these exceptions, an overall preference of PPEP-1 for a Pro at the P3′ was observed (Figure 4A). The ability of PPEP-1 to hydrolyze substrates with His, Phe, and Tyr at P3′ might be the result of aromatic–aromatic interactions (π–π stacking) with the Trp-103 and these residues.^45^ In this scenario, a Pro residue at the P2′ position is probably necessary to redirect the substrate from the diverting loop.

For PPEP-2, much less was known about the prime-side specificity because the initial identification of its cleavage site (PLPPVP) was based on the similarity in genomic organization of PPEP-1 and -2 and their endogenous substrates.^10^ To a certain extent, PPEP-2 showed overlapping specificity with PPEP-1 (Figure 4B). For example, a high level of the PPPGGLEEF peptide was found and PPEP-2 also allows PPH at the P1′-P3′ positions. However, in line with the endogenous substrate (Figure 1), PPEP-2 prefers a valine at the P2′ (Figure 4B). Moreover, in contrast to PPEP-1, not all optimal substrates for PPEP-2 had at least two prolines at their P1′-P3′ positions. Of note, all peptides without prolines at the P2′ and P3′ positions had a Val at the P2′ position (Figure 4B), again indicating that this is a strong determinant for PPEP-2 susceptibility (Figure 1).

As mentioned above, we demonstrated for the first time that PPEP-3 is a genuine PPEP that cleaves Pro-Pro bonds (Figure 4C). For PPEP-3, the most abundant product peptide corresponded to PPPGGLEEF (Figure 4C). Since this peptide was relatively much more abundant than peptides with other amino acids at the P2’ and P3′ positions, this resulted in an overall motif that was dominated by proline at the P1′-P3′ positions. Still, PPEP-3 allowed several other residues at the P3′ that were not tolerated by the other two PPEPs. Furthermore, unlike the other PPEPs, PPEP-3 was able to cleave a PPHP motif (P1-P3′), as represented by the PHPGGLEEF product peptide (Figure 4C).

**Figure 5:**
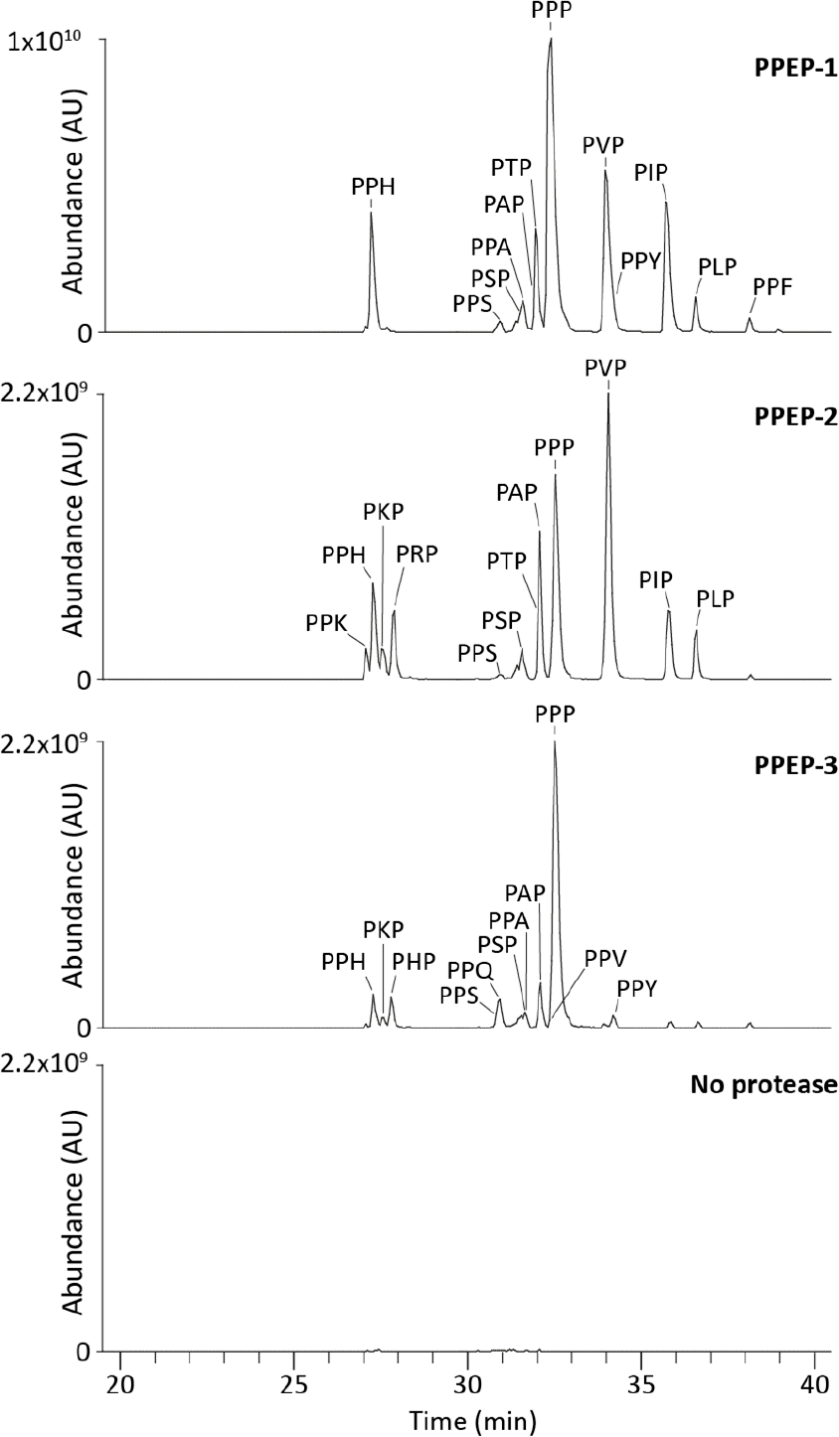
Extracted ion chromatograms of PXP(GGLEEF)/PPX(GGLEEF) product peptides after incubation with PPEPs reveal prime-side specificity profiles. The full combinatorial peptide library was incubated with each of the PPEPs for 3 h. A nontreated control was included to identify the amount of background peptides. After analysis of the product peptides using LC-MS/MS, EICs were constructed for all possible PXP/PPX product peptides (in total 19, both 1+ and 2+ *m*/*z* values were used). Discrimination between PXP and PPX peptides relied on both inspection of fragmentation spectra and separation on a C18 column (Figures S3 and S4). If product peptides were not separated on the column, lines indicate the relative abundances of the nonseparated peptides. Mass tolerance was set to 10 ppm.

Collectively, the above results showed that all three PPEPs preferred at least one proline at the P2′ or P3′ position. To emphasize the differences in such product peptides, extracted ion chromatograms (EICs) of every possible PXPGGLEEF/PPXGGLEEF peptide were constructed (Figure 5). Not only does this clearly show the difference in product profiles, but it also reveals the differences between PXP and PPX peptides such as PHP and PPH.

To test the reproducibility of our method, we performed three additional replicate experiments with all three PPEPs. The results from these experiments show excellent reproducibility (Figure S6). Moreover, the overall profiles of the PXPGGLEEF/PPXGGLEEF peptides look very similar to those presented in Figure 5.

Although in the current design our library is primarily suitable to investigate PPEPs, other proteases that can cleave between the two “XX” sequences in the library peptides could also be tested, assuming that their activity is not compromised by the presence of the surrounding prolines. However, we anticipate that for other proteases, a different library design would be beneficial, while still using the same central concept of our approach. For example, the addition of the GGLEEF tail as used in our library can be easily translated to other libraries as well. Although for the current experiments with the PPEPs we used a library with two fixed positions, we believe that a strategy using randomization at five sites with only one fixed position would still be possible and provide a broad understanding of the subsite specificity. However, due to the OBOC principle,^30^ not all individual peptides (2.4 million options when using 19 amino acids) will be present in such a library when starting with the same number of beads as used for our current synthesis (approximately 1.000.000). Although our experiments with PPEP-1 and the two P3-sublibraries showed that partial information about the nonprime-side specificity can also be obtained with our method, we believe that a complementary XXPPXX library, in which the biotin is attached to the C-terminus of the peptides, is essential for a more comprehensive characterization of the nonprime-side specificity. Since the negative selection for substrates proceeds identically to that of the current library, both libraries can be mixed, allowing for the profiling of both the prime-side as well as the nonprime-side in a single experiment.

### Incubation of PPEP-1 with a Collection of FRET-Quenched Substrate Peptides Confirms Its Preference for Different Amino Acids at the P2′ Position

**Figure 6:**
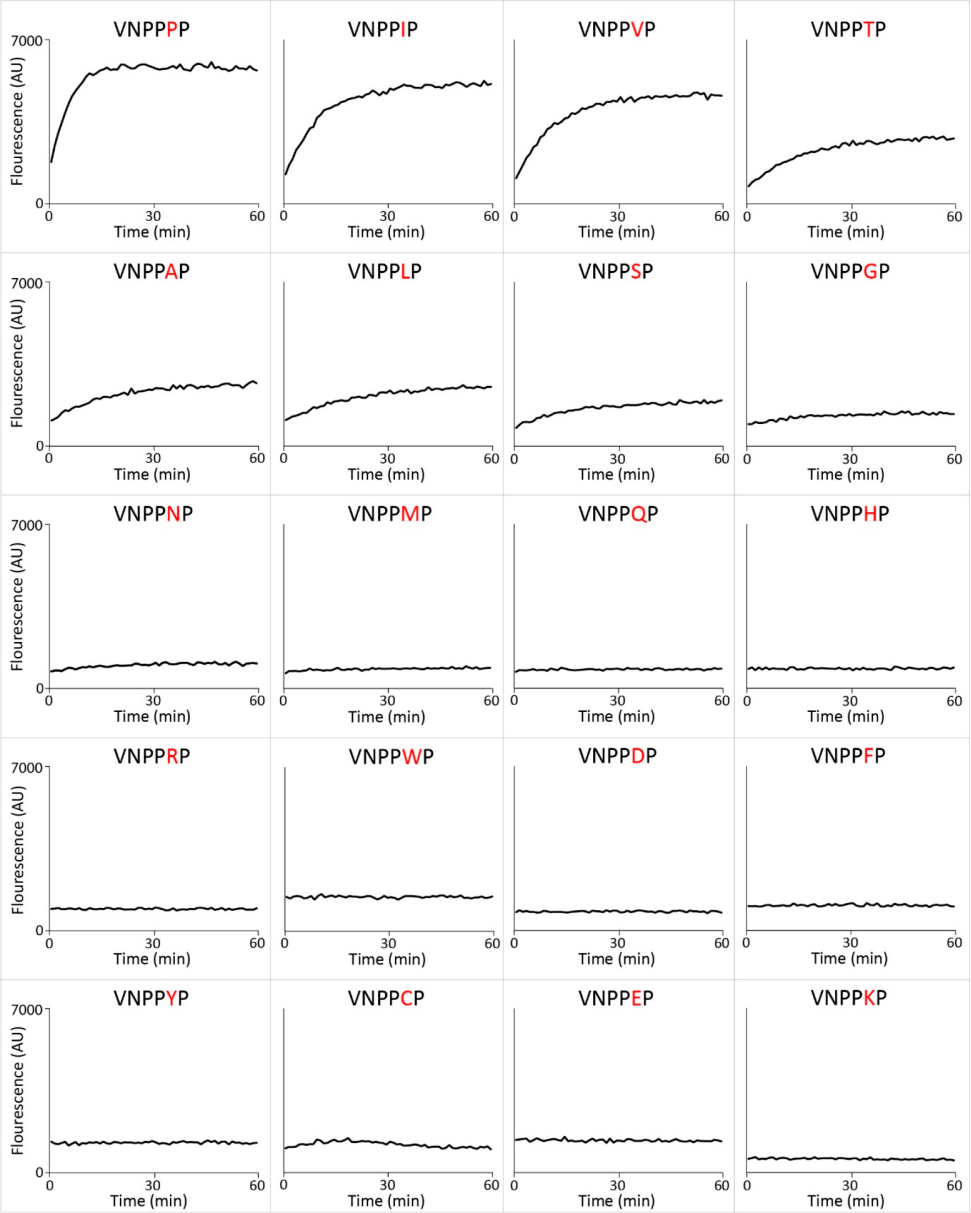
Time course of PPEP-1 mediated cleavage of synthetic FRET-quenched peptides with permutations at the P2′ position. The PPEP-1 substrate peptide VNP↓PVP was permutated to generate FRET-quenched peptides (Lys_Dabcyl_-EVNPPXPD-Glu_Edans_) containing any of the standard 20 amino acids at the P2′ position. These peptides were incubated with PPEP-1 and fluorescence was measured during 1 h. Peptides are sorted from the top left to bottom right based on their cleavage efficiency.

Based on the endogenous substrates (Figure 1) and a small synthetic peptide library,^11^ PPEP-1 was expected to only tolerate V, I, A, and P at the P2′ position. To substantiate our results with the combinatorial peptide library, we synthesized 20 PPEP-1 FRET-quenched substrate peptides that only differed at the P2′ position (Lys_Dabcyl_-EVNP↓PXPD-Glu_Edans_) and tested these with PPEP-1 in a time course kinetic assay. The results of these experiments are depicted in Figure 6, in which substrates are ranked (from top left to bottom right) based on their increase in fluorescence during the 1 h incubation. Overall, these data (Figure 6) correlated well with the results of the combinatorial library experiment (Figure 5). Although cysteines were not included in the combinatorial library design (Figure 2), the results with the VNPPCP FRET-peptide showed that it is not tolerated at the P2’ position by PPEP-1.

### PPEP-3 is Able to Cleave Endogenous PPEP-1 and PPEP-2 Substrates when the Valine at the P2′ Position is Replaced by a Proline

**Figure 7:**
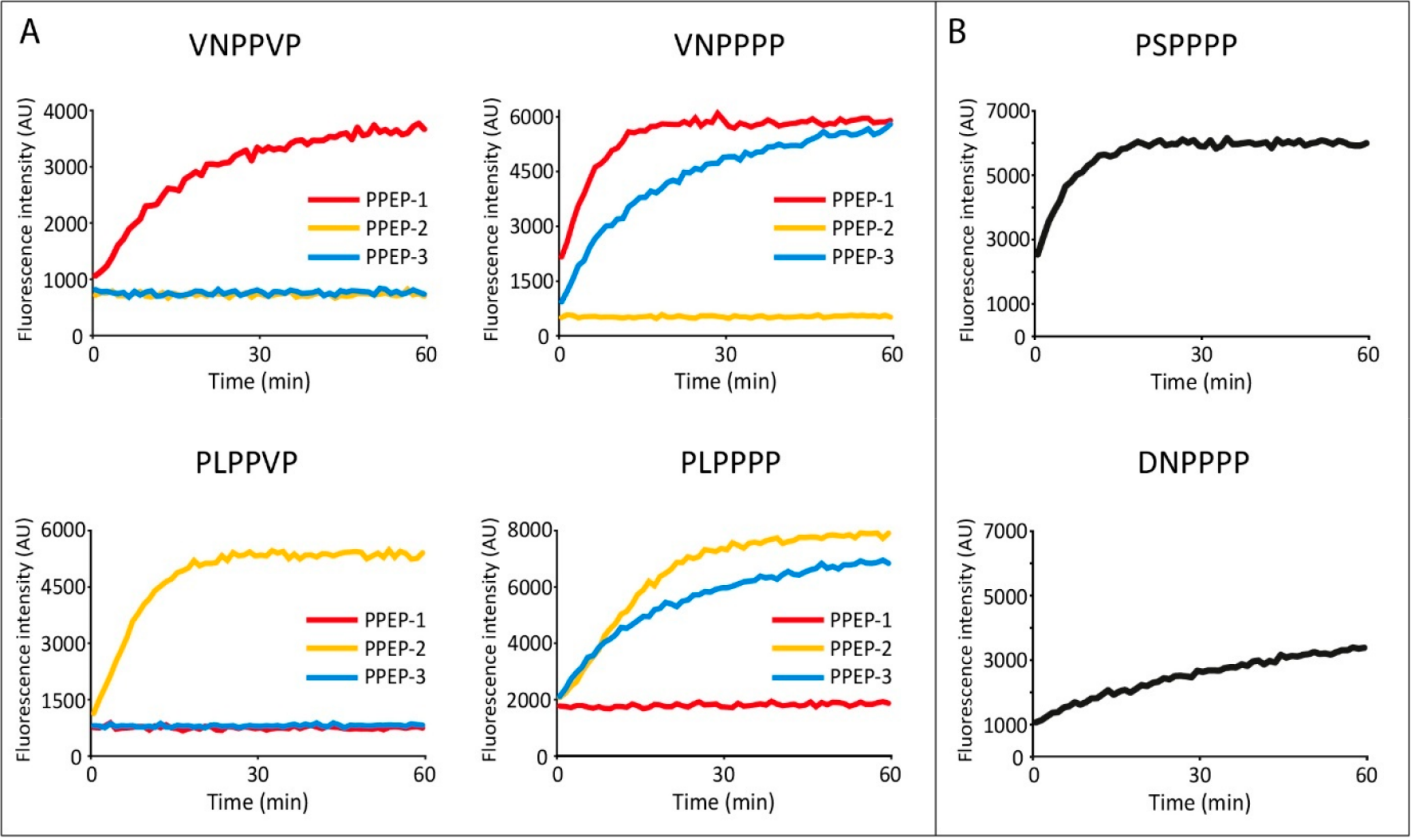
Time course of cleavage of synthetic FRET peptides by PPEP-1, PPEP-2, and PPEP-3. (A) Cleavage of PPEP-1 (Lys_Dabcyl_-EVNP↓PVPD-Glu_Edans_) and PPEP-2 (Lys_Dabcyl_-EPLP↓PVPD-Glu_Edans_) substrate peptides, and their P2′ = Pro variants, by PPEP-1, PPEP-2, and PPEP-3. (B) Cleavage of peptides containing cleavage motifs from putative *G. thermodenitrificans* PPEP-3 substrates by PPEP-3. Only the core sequences (P3-P3′) of the individual FRET-quenched peptides are indicated.

The endogenous substrates of PPEP-1 and PPEP-2 contain the PVP motif at P1′-P3′ (Figure 1) and the corresponding product peptides (PVPGGLEEF) were clearly observed using the combinatorial library approach (Figure 5). However, this product peptide was not observed with PPEP-3 (Figure 5), indicating that the corresponding PPEP-1 and PPEP-2 substrate peptides are most likely not cleaved by PPEP-3. We tested this hypothesis using two synthetic FRET-quenched substrate peptides, i.e., Lys_Dabcyl_-EVNPPVPD-Glu_Edans_ and Lys_Dabcyl_-EPLPPVPD-Glu_Edans_, representing substrates of PPEP-1 and PPEP-2, respectively (Figure 1). In line with our expectations, PPEP-3 did not hydrolyze either peptide (Figure 7A). However, when the P2′ Val of both peptides was replaced by a Pro, cleavage by PPEP-3 did occur (Figure 7A). On the contrary, although PPEP-1 and PPEP-2 can cleave peptides with four prolines at the P1-P3′ position (Figures 4A,B and 5), they can still not cleave each other’s substrate when the Val at the P2′ position is replaced by a proline (Figure 7A).

The high specificity of each of the PPEPs for amino acids surrounding the Pro-Pro motif remains obscure. Remarkably, based on the amino acid residue at position 103 (Trp-103) in PPEP-1, two groups were distinguished.^12^ In addition to PPEP-1, the Trp-103 group includes PPEP-2. The other group, to which PPEP-3 belongs, has a Tyr at this position (Figure S7). Interestingly, a PPEP-1 W103Y mutant showed very low activity toward a substrate peptide as compared to WT.^46^ For PPEP-2, the importance of this residue has been less explored. Nevertheless, our data with PPEP-3 show that a tyrosine at this position is compatible with PPEP activity. Whether the tyrosine in PPEP-3 that corresponds to the Trp-103 in PPEP-1 (Tyr-112, Figure S7) is responsible for the difference in prime-side specificity between PPEP-3 and the other two PPEP-s requires structural information, especially of a substrate-bound cocrystal.

### Peptides with an XXPPPP Motif, as Observed in *Geobacillus thermodenitrificans* Proteins are Cleaved by PPEP-3

Next, we looked for possible endogenous substrates of PPEP-3. For PPEP-1 and PPEP-2, genes encoding their substrates are found adjacent to the protease gene (Figure 1). Next to PPEP-3, a gene encoding a protein (YpjP, Figure 1) with three XXPPXX sequences is found (VTPPAS, EHPPQD, and NTPPNW). In line with the data from the combinatorial library, the corresponding FRET-quenched peptides were not cleaved by PPEP-3 (data not shown). Overall, our data from the library experiment indicate a strong preference of PPEP-3 for all prolines at the P1-P3′ positions (Figures 4C, 5, and 7A). Based on this observation, we hypothesized that possible endogenous substrates containing an XXP↓PPP motif are present in *G. thermodenitrificans* strain NG80-2. Indeed, *G. thermodenitrificans* encodes for four proteins containing four consecutive prolines, two of which contain a signal peptide for secretion as determined by DeepTMHMM and SignalP 6.0 (Figure S8).^47,48^ This last feature is thought to be of importance since PPEP-3 itself is predicted to be a secreted protein. One of the identified proteins, GTNG_0956, contains both a putative CAP-domain and an SCP-domain. Admittedly, signal peptide prediction by SignalP 6.0 is inconclusive for this protein, since the signal peptide would be short in length and no cleavage site is predicted (Figure S8B). In contrast, DeepTMHMM predicts a signal peptide with a higher confidence (Figure S8C). The other protein with an XXPPPP motif and a signal peptide is GTNG_3270. This protein is predicted with high confidence to possess a Sec/SPII signal sequence for integration in the lipid membrane. However, no functional domains were found for this protein. The putative PPEP-3 cleavage sites in GTNG_0956 and GTNG_3270 are PSP↓PPP and DNP↓PPP, respectively. We tested synthetic FRET-quenched peptides containing these motifs for cleavage by PPEP-3 (Figure 7B). Both FRET peptides were indeed cleaved by PPEP-3, with PSPPPP being the optimal substrate of the two. MALDI-ToF MS analysis confirmed the cleavage between the two prolines within these peptides (Figure S9). Collectively, the above data show that the results from the library experiment resulted in testable hypotheses about possible endogenous PPEP-3 substrates in *G. thermodenitrificans* strain NG80-2. For PPEP-1 and PPEP-2, the endogenous substrates were identified based on synthetic peptides, bioinformatic predictions, and MS-based secretome analyses.^10,36^ Interestingly, none of the sites in the endogenous substrates of these two PPEPs has four consecutive prolines, even though for both proteases the PPPGGLEEF product peptide was (one of) the major product peptides. In order to identify the endogenous substrate of PPEP-3, additional experiments such as secretome analyses in combination with gene knockout studies are needed, although we cannot exclude the possibility that the substrate originates from a different organism than *G. thermodenitrificans*.

## Conclusion

In conclusion, we show for the first time a strategy to study the activity and specificity of a protease by combining a combinatorial synthetic peptide library with mass spectrometry. Our method takes each amino acid into account (with the exception of cysteine) and directly showed combinations of amino acids that were tolerated at the P2′ and P3′ positions. We believe that the strategy presented here is a generic one that can, with a tailored design of the library, also be used to explore substrate specificities of other proteases. Importantly, with the new method we have not only confirmed the prime-side specificity of PPEP-1 and PPEP-2, but also revealed some new unexpected peptide substrates. Moreover, we have characterized a new PPEP (PPEP-3 from *Geobacillus thermodenitrificans*) that has a prime-side specificity that is very different from that of the other two PPEPs.

## Data Availability

The mass spectrometry proteomics data have been deposited to the ProteomeXchange Consortium^49^ via the PRIDE^50^ partner repository with the data set identifier PXD038277.

## Supporting Information Available

Supplementary methods; Fragmentation spectrum of PYVGGLEEF (Figure S1); Chromatographic and MS/MS fragmentation characteristics of peptides KYGGLEEF, YKGGLEEF, and PPPGGLEEF (Figure S2); Fragmentation spectra of product peptides (Figure S3); Separation of product peptides on C18 column (Figure S4); FRET-quenched peptide cleavage assay (Figure S5); Data from three independent incubations of the full peptide library with PPEP-1, -2, and -3 (Figure S6); Alignment of PPEPs (Figure S7); signal peptide prediction of putative PPEP-3 substrates (Figure S8); MALDI-ToF MS analysis of the product peptides from the incubations of PPEP-3 with two FRET-peptides (Figure S9) (PDF)
Database search of LC-MS/MS data (XLSX)
