# Supplementary material for: In-Depth Specificity Profiling of Endopeptidases Using Dedicated Mix-and-Split Synthetic Peptide Libraries and Mass Spectrometry
Source: Anal Chem. 2023 Jul 26;95(31):11621–31. doi: 10.1021/acs.analchem.3c01215 (PMC10413326; doi:10.1021/acs.analchem.3c01215)
Supplement: Supplementary file 1 — ac3c01215_si_001.pdf [file ac3c01215_si_001.pdf]

Supporting information

In-depth specificity profiling of endopeptidases using dedicated mix-and-split synthetic peptide libraries and mass spectrometry

Bart Claushuis[1], Robert A. Cordfunke[2], Arnoud H. de Ru[1], Annemarie Otte[1], Hans C. van Leeuwen[3], Oleg I. Klychnikov[4], Peter A. van Veelen[1], Jeroen Corver[5], Jan W. Drijfhout[2], Paul J. Hensbergen[1*]

[1] Center for Proteomics and Metabolomics, Leiden University Medical Center, Leiden, 2333 ZA, The Netherlands

[2] Department of Immunology, Leiden University Medical Center, Leiden, 2333 ZA, The Netherlands

[3] Department of CBRN Protection, Netherlands Organization for Applied Scientific Research TNO, Rijswijk, 2280 AA, The Netherlands

[4] Department of Biochemistry, Moscow State University, Moscow, 119991, Russian Federation

[5] Department of Medical Microbiology, Leiden University Medical Center, Leiden, 2333 ZA, The Netherlands

*Correspondence to:

P.J. Hensbergen

Center for Proteomics and Metabolomics

Leiden University Medical Center

PO Box 9600

2300 RC Leiden

The Netherlands.

Tel.: +31-71-5266394

Fax: +31-71-5266907

E-mail: P.J.Hensbergen@lumc.nl

Contents

Expression and purification of PPEPs S3

Synthesis of the combinatorial peptide library S3

MALDI-FT-ICR MS S3

FRET peptide cleavage assays S4

Bioinformatic analyses S4

Figure S1. Fragmentation spectrum of PYVGGLEEF S5

Figure S2. Chromatographic and MS/MS fragmentation characteristics of peptides KYGGLEEF, YKGGLEEF and PPPGGLEEF. S6

Figure S3. Synthetic PXP/PPX product peptides display distinct fragmentation spectra. S7

Figure S4. Separation of PXP/PPX peptides on C18 column S8

Figure S5. PPEP-1 can cleave a VNPPPH peptide but not a VNPPHP peptide S9

Figure S6. Extracted ion chromatograms of PXPGGLEEF/PPXGGLEEF product peptides from three independent incubations of the full peptide library with PPEP-1, -2 and -3 .... S10

Figure S7. Alignment of PPEP-1, PPEP-2 and PPEP-3 .... S11

Figure S9. MALDI-ToF MS analysis of the product peptides from the incubations of PPEP-3 with the two FRET-peptides as presented in Figure 7B S13

References S14

Expression and purification of PPEPs

PPEP-1 and PPEP-2 were expressed and purified as previously described[1,2]. For the expression of PPEP-3, a pET28a vector containing an *E. coli* codon optimized 6xHis-PPEP-3 (lacking the signal peptide) construct was ordered from Twist Bioscience. The pET-28a 6xHis-PPEP-3 plasmid was transformed to *E. coli* strain Rosetta and PPEP-3 expression was induced using 1 mM IPTG. Lysates were prepared as described in the protocol for preparation of cleared *E. coli* lysates under native conditions as described in the fifth edition of the QIAexpressionist (Qiagen). The lysates were loaded onto a 1 ml HisTrap HP column (GE healthcare) coupled to an ÄKTA Pure FPLC system (GE healthcare). Column was washed using wash buffer (50 mM NaH_2PO_4, 300 mM NaCl, 20 mM imidazole) and 6xHis-PPEP-3 was eluted using a step gradient with elution buffer (50 mM NaH_2PO_4, 300 mM NaCl, 500 mM imidazole). Imidazole was removed by dialysis using 50 mM NaH_2PO_4, 300 mM NaCl.

Synthesis of the combinatorial peptide library

Combinatorial peptide libraries were synthesized basically as has been previously described[3]. In short, peptide libraries were synthesized by solid phase peptide synthesis on a Syro II peptide synthesizer (Multisyntech, Germany). Synthesis was performed in 19 reactors (2 ml) using about 1 g of Tentagel resin (Rapp-polymere, Germany) resin (total loading 190 µmol), applying Fmoc chemistry with HATU/NMM activation, 20 % piperidine in NMP for Fmoc removal and NMP as a solvent. For each fixed position in each reactor the same amino acid was coupled, for each random position (X) in each reactor a different amino acid was coupled, after which the resin beads were removed from each reactor, mixed thoroughly, and equally split over the 19 reactors again to allow for the subsequent stages of the synthesis. After the last random position, the resin beads were not mixed, leaving 19 sub-libraries. Biotin was introduced into the resin bound peptides by a two hour coupling with a sixfold equimolar preactivated mixture of biotin and PyBop. Cleavage using TFA/water/ethanethiol 18/1/1, 3h, RT, was used to isolate the peptides from the resin. Approx. 12 ml ether/pentane was added to each sub-library and sub-libraries were incubated at -20 °C for 10 min before centrifugation at 3300 rpm for 10 min at -9 °C. Pellets were washed with approx. 13 ml ether/pentane and air-dried. Dried pellets were resuspended in 2 ml MilliQ/acetonitrile and freeze-dried. Stocks of 10 nmol peptide/µl were prepared in DMSO.

MALDI-FT-ICR MS

To analyze samples using MALDI-FT-ICR MS, the vacuum dried product peptides were reconstituted in 20 µl 0.1% formic acid. Next, 1 µl sample was combined with 1 µl matrix (5 mg/ml α-Cyano-4-

hydroxycinnamic acid) and 1 µl was spotted on an AnchorChip target (Bruker). Analysis was performed on a 15 T MALDI-FT-ICR MS (Bruker Daltonics).

FRET peptide cleavage assays

Time course kinetic experiments with PPEPs were performed using fluorescent FRET-quenched peptides. FRET peptides consisted of Lys_{Dabcyl}-EXXPPXXD-Glu_{Edans}, in which X varied between the different peptides tested. To test cleavage of FRET peptides by PPEPs, 75 µL of FRET peptide (100 µM in PBS) was added to a well of a 96-well Cellstar black plate (Greiner). Immediately prior to the assay, 75 µl PBS containing a PPEP (0.2-1 µg) was added. Peptide cleavage was measured using the Envision 2105 Multimode Plate Reader. Fluorescence intensity was measured each minute for 1 h, with 10 flashes per measurement. The excitation and emission wavelengths were 350 nm and 510 nm, respectively. When comparing PPEP-1, PPEP-2, and PPEP-3 in a single experiment, the relative fluorescence was determined by regarding the highest signal as 100%.

Bioinformatic analyses

PPEP-3 structure prediction was carried out using Colabfold [4] with the following parameters: template mode=none, MSA mode=MMseqs2, pair mode=unpaired+paired, model type=auto, and number of recycles=3. Signal peptide predictions were performed using DeepTMHMM[5] and SignalP 6.0[6]. For sequence alignments, the Clustal Omega Multiple Sequence Alignment tool was used[7].

The cleavage motifs were created using Weblogo 3[8] with the units set to probability. The sequences logos were generated based on the relative intensities of the 10 most abundant product peptides for each PPEP. A list with these 10 product peptides was created, in which each individual peptide occurred a number of times, according to its relative abundance to the other peptides. For example, if product ‘A’ was 100 times more abundant than product ‘B’, product ‘A’ was present 100 times more in this list than product ‘B’.

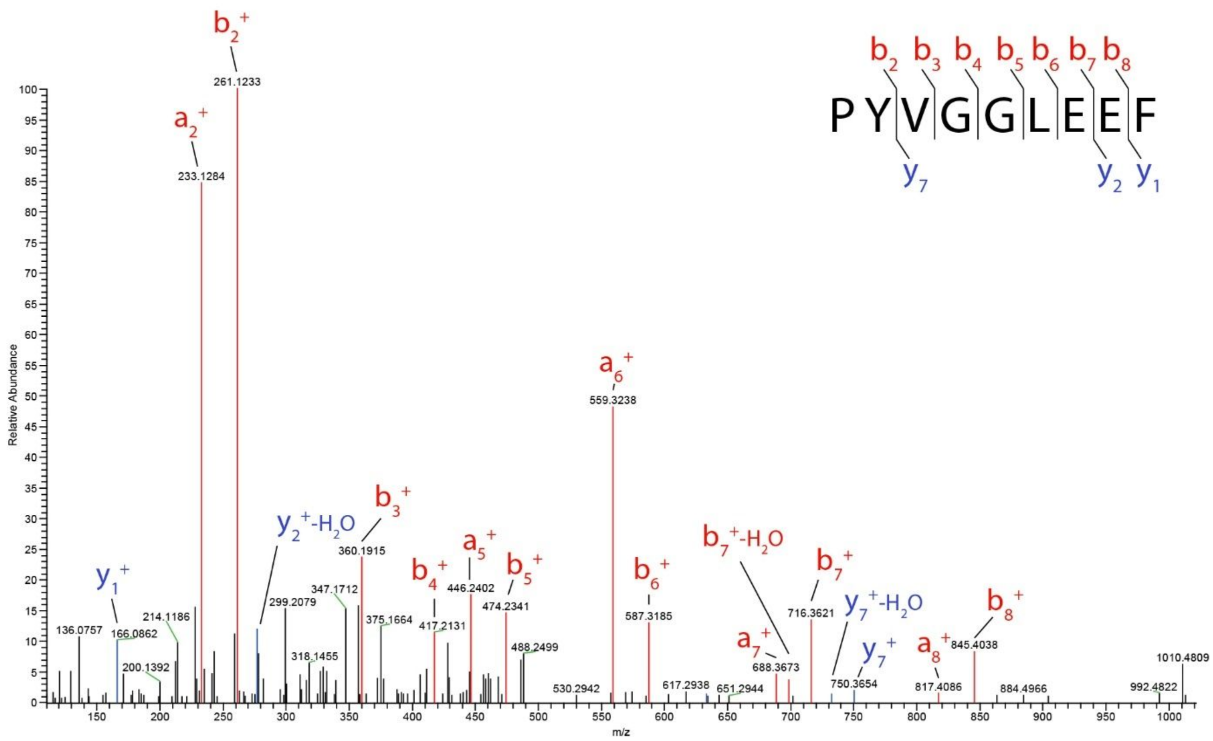

Figure S1. Fragmentation spectrum of PYVGGLEEF. MS/MS spectrum of the PYVGGLEEF peptide that was used in the design of the combinatorial peptide library.

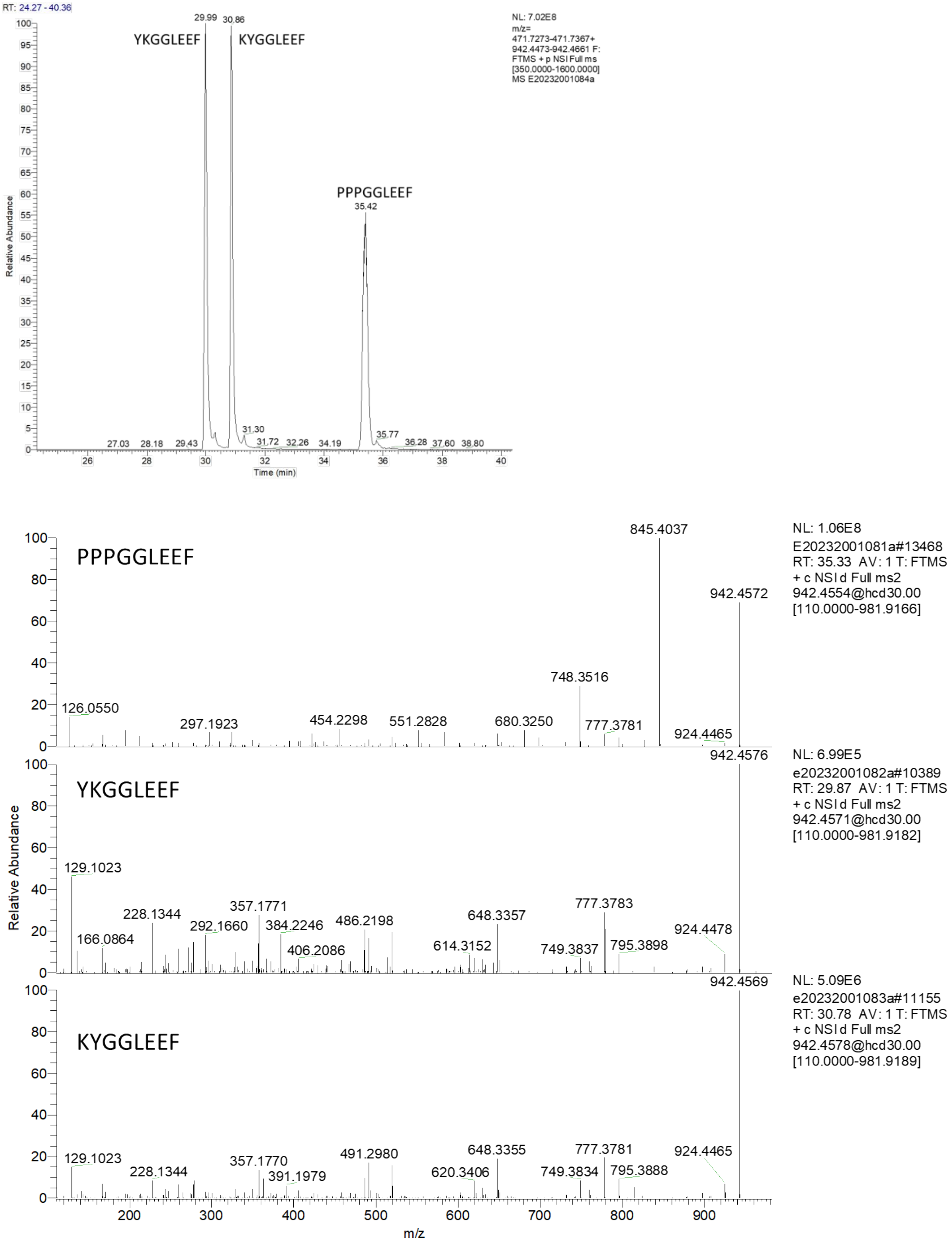

Figure S2. Chromatographic and MS/MS fragmentation characteristics of peptides KYGGLEEF, YKGGLEEF and PPPGGLEEF. Synthetic peptides KYGGLEEF, YKGGLEEF and PPPGGLEEF (1 uL of a 1:1:1 (v/v/v) mix of 200 fmol/uL of each peptide) were analyzed using LC-MS/MS. Upper panel: chromatographic behavior. Peaks are assigned based on injections of individual peptides. Lower panel: MS/MS spectra.

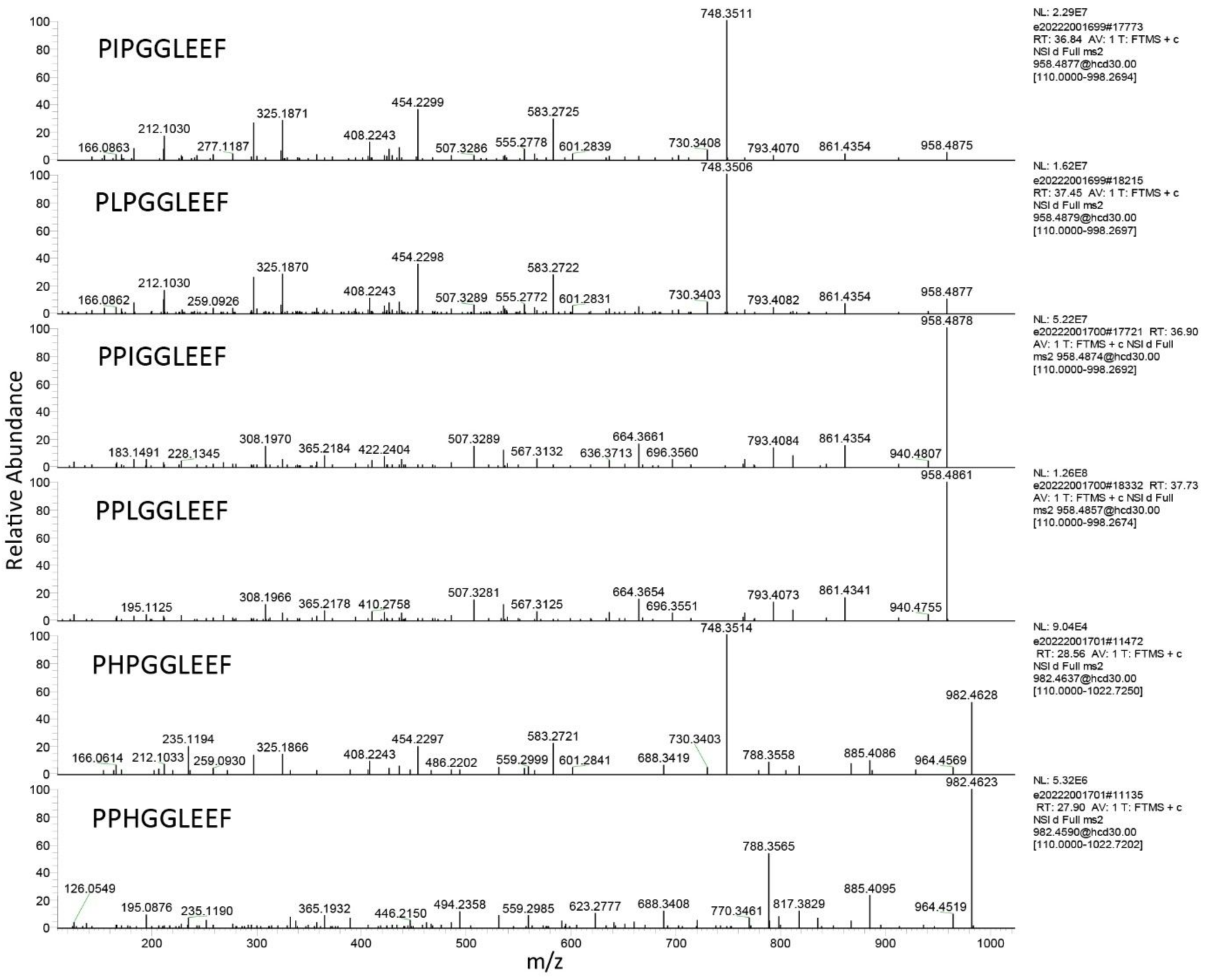

Figure S3. Synthetic PXP/PPX product peptides display distinct fragmentation spectra. Synthetic peptides with either a PXP or PPX motif were analyzed using LC-MS/MS.

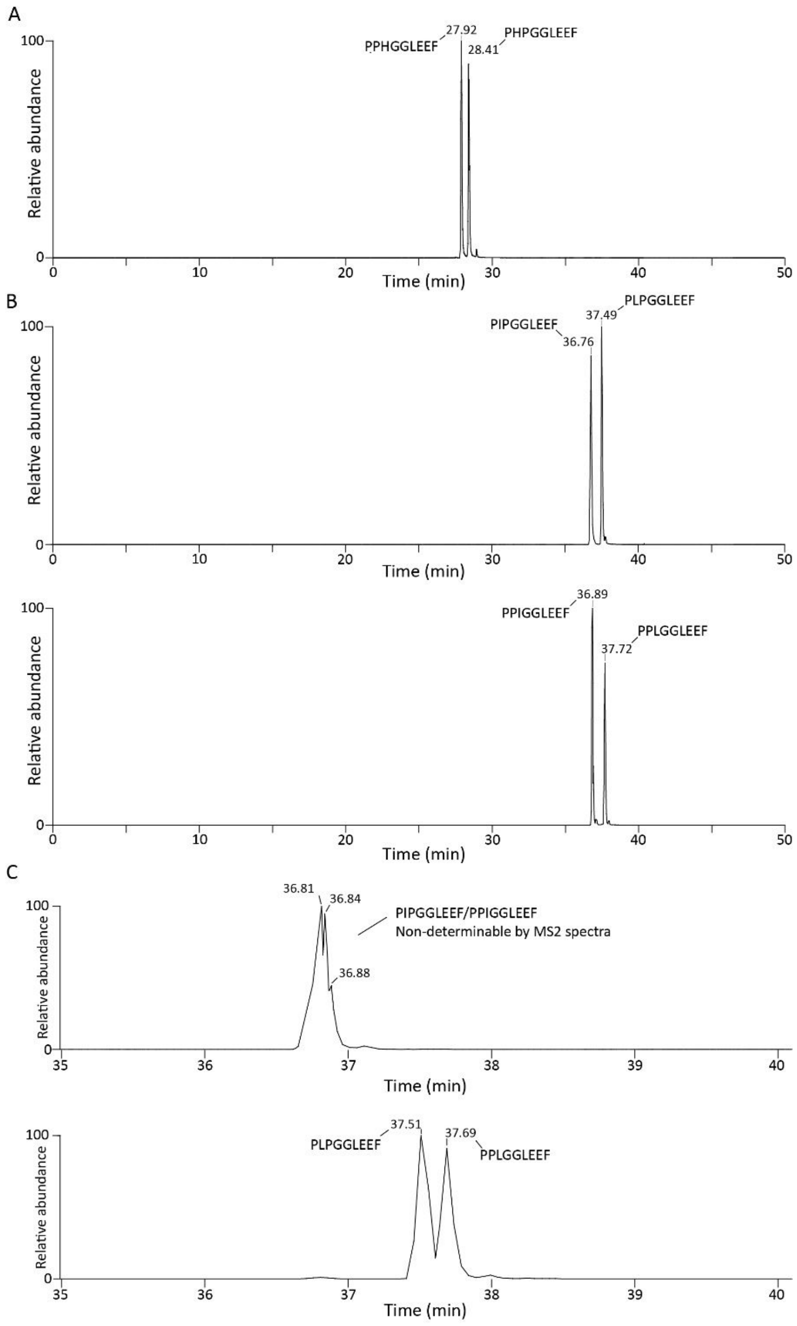

Figure S4. Separation of PXP/PPX peptides on C18 column. Shown are extracted ion chromatograms of the corresponding peptides with a mass tolerance of 10 ppm. **A)** Synthetic peptides PHPGGLEEF and PPHGGLEEF were mixed at an equimolar concentration and analyzed using LC-MS/MS. Assignment of the peaks is based on LC-MS/MS analyses of the two peptides separately (data not shown). **B)** PXP/PPX peptides that contain either a Leu or Ile residue are separated on a C18 column. Assignment of the peaks is based on LC-MS/MS analyses of the two peptides separately (data not shown). **C)** PIPGGLEEF and PPIGGLEEF are not fully separated on a C18 column. Although some separation is observed, no conclusions could be made about the order of elution. However, panel B shows a lower retention time for PIPGGLEEF. PLPGGLEEF and PPLGGLEEF are, although not completely, separated on the column.

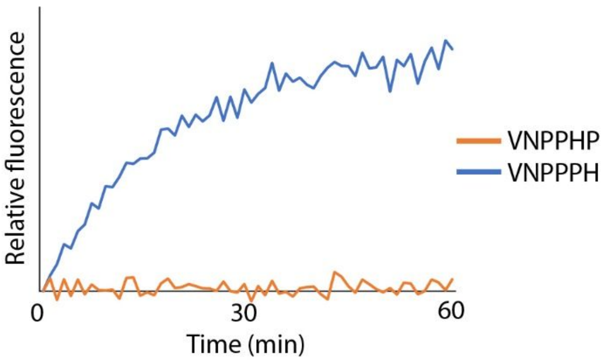

Figure S5. PPEP-1 can cleave a VNPPPH peptide but not a VNPPHP peptide. FRET-quenched peptides containing either VNPPHP or VNPPPH were incubated with PPEP-1 for 1 h. Fluorescence was measured in a fluorescence microplate reader.

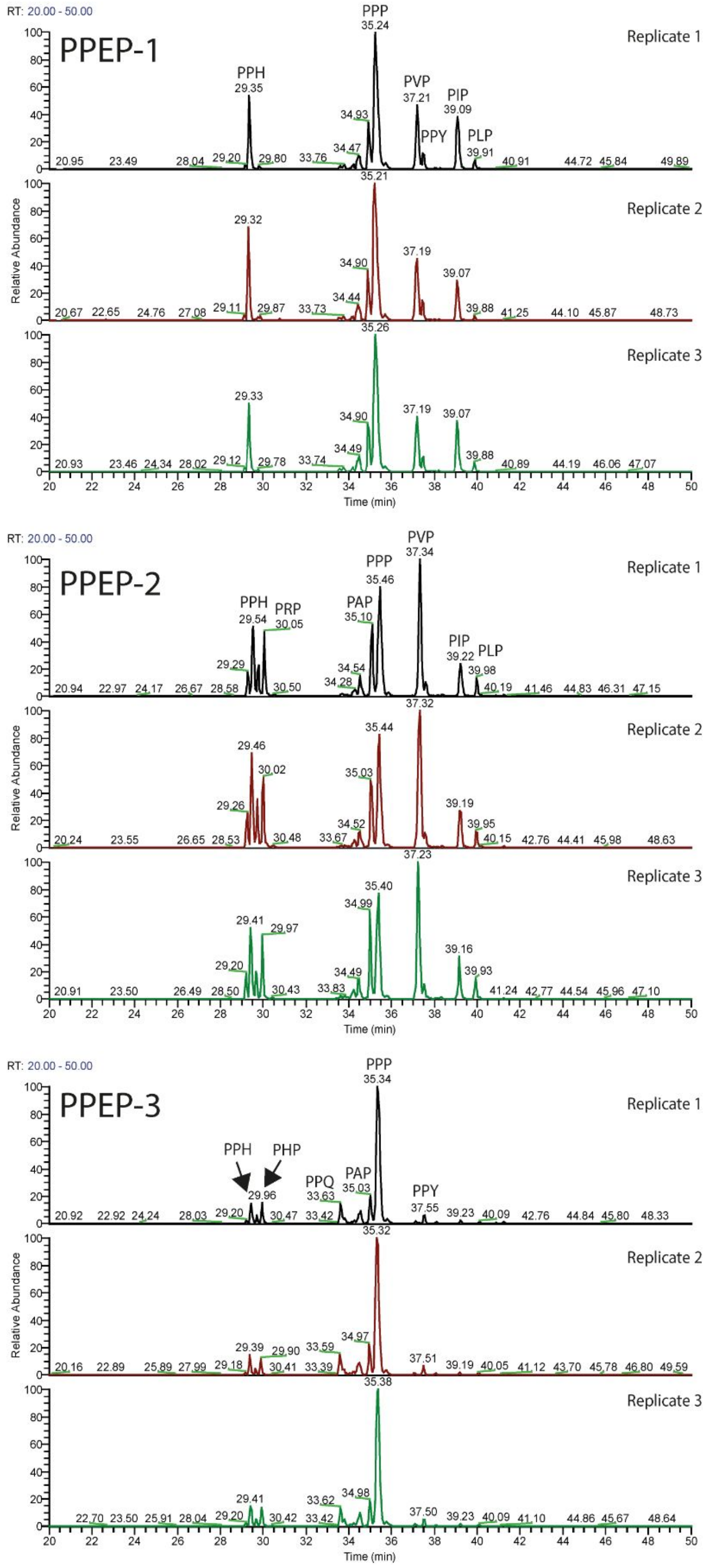

Figure S6. Extracted ion chromatograms of PXP/PPX product peptides from three independent incubations of the full peptide library with PPEP-1, -2 and -3. See also Figure 5.

```
PPEP-1      -MRPSKKLLIAIISIFLISSVPVSAH--------ADSTTIQQNKDTLSQIVVFPTGNYDK      51
PPEP-2      -MKWDKRVVALILAVMIVCP--L-----------FAAPAHAQEQSILDKLVVLPSGEYNH      46
PPEP-3      MRRLLTSLLAALLAVPLLSLSPYPAAHGVLLEESSLDVQTIPSHDVLGRIVIVPETDFSF      60
              :  . ::  :::: ::.                       .:. *.::*:.*  ::.

                                                          Trp-103
PPEP-1      NEANAMVNRLANIDGKYLNALKQNNLKIKLLSGKLTDEKEYAYLKGVVPKGWEGTGKTWD      111
PPEP-2      SEAAAMKQRLEKIPTSILDALYSKGVKIKLTQGAITNEPELAYLKGVVPRGWEGTGLTWD      106
PPEP-3      DEANEMIRTLARIDRRILEQAANHHIYIQLLTNPITDEPIARHLRGKTPRGYVPGSKTWD      120
            .**  * . * .*    *:   .: : *:*  . :*:*    :*:* .*:*:   . ***

PPEP-1      DVPGLGGS-TVALRIGFSNKGKGHDAINLELHETAHAIDHIVLNDISKSAQFKQIFAKEG      170
PPEP-2      DVPGVSER-VVAVRIGYSEKGKGHNSLNLEIHETLHAVDRLVLNEVSGTDEFINIFNKEA      165
PPEP-3      EVPGIGGAHLVLVRLGHSEKGKGHGSINLELHEFAHSLDYIVFDHIHETDEFQALWREEA      180
            :***:.    * :*:*.*:*****.::***:**  *::* :*::.:  : :*  :: :*.

PPEP-1      R-SLGNVNYLGVYPEEFFAESFAYYYLNQDTNSKLKSACPQTYSFLQNLAK---      220
PPEP-2      SVKYKGDGYVSAYPTEYFAEAASLYLYSDATRSDLKDSMPLTYEFMAKLFAN--      217
PPEP-3      PQLFPREYYFLTYPEEYFAESFAYYYVSEKTQETLRMAAPRTYTFIRQLAERAS      234
```

Figure S7. Alignment of PPEP-1, PPEP-2 and PPEP-3. Alignment was created using the Clustal Omega multiple sequence alignment tool.

A

Protein	Gene name	Predicted PPEP-3 cleavage motif
Uncharacterized protein	GNTG_0956	PSP↓PPP
Uncharacterized protein	GTNG_1059	TAP↓PPP
Uncharacterized protein	GTNG_1937	VDP↓PPP
Uncharacterized protein	GTNG_3270	DNP↓PPP

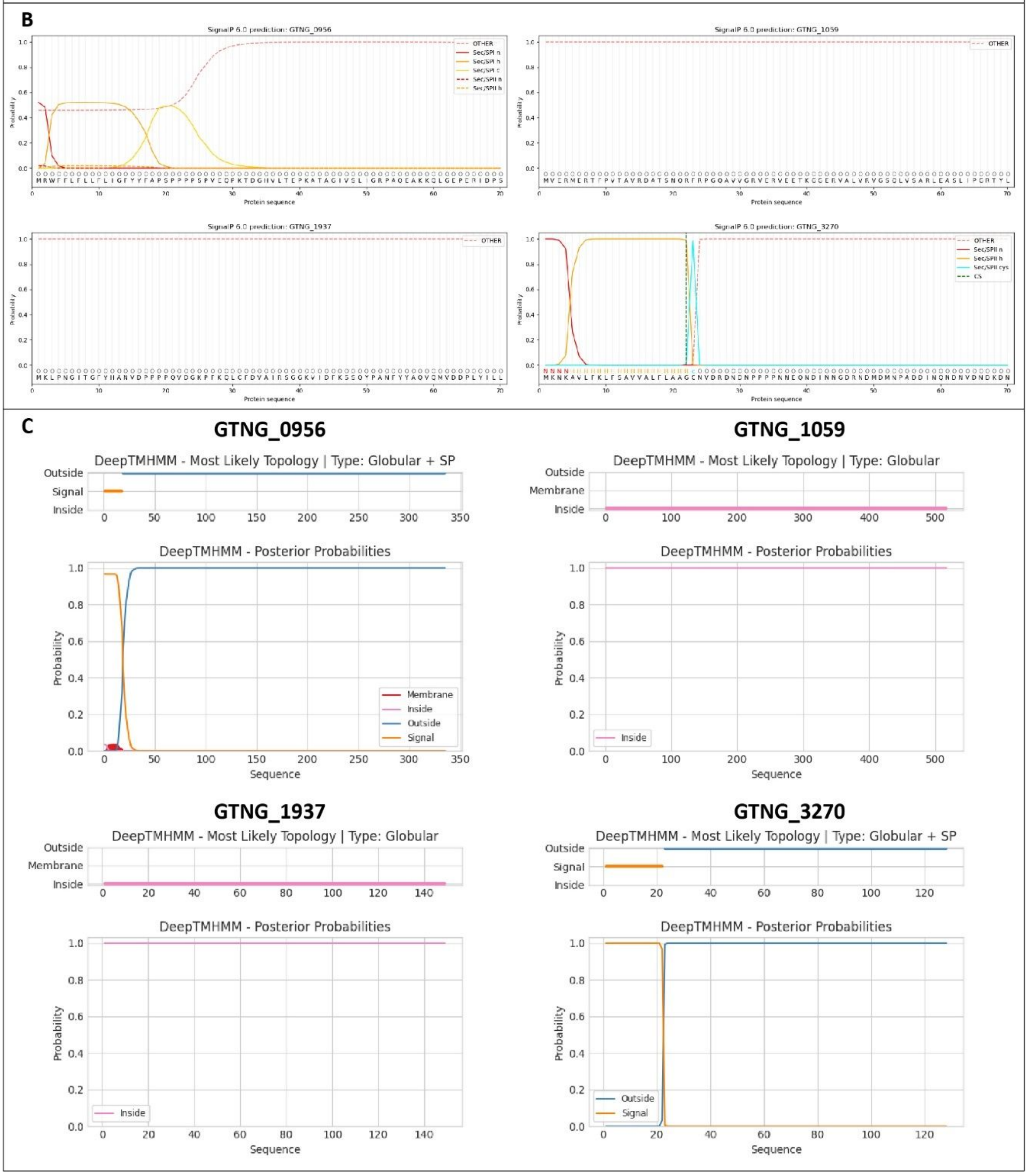

Figure S8. Signal peptide prediction of putative PPEP-3 substrates in G. thermodenitrificans. a Overview of the proteins and their predicted PPEP-3 cleavage motifs. **b** Signal peptide prediction by SignalP 6.0. **c** Signal peptide prediction by DeepTMHMM.

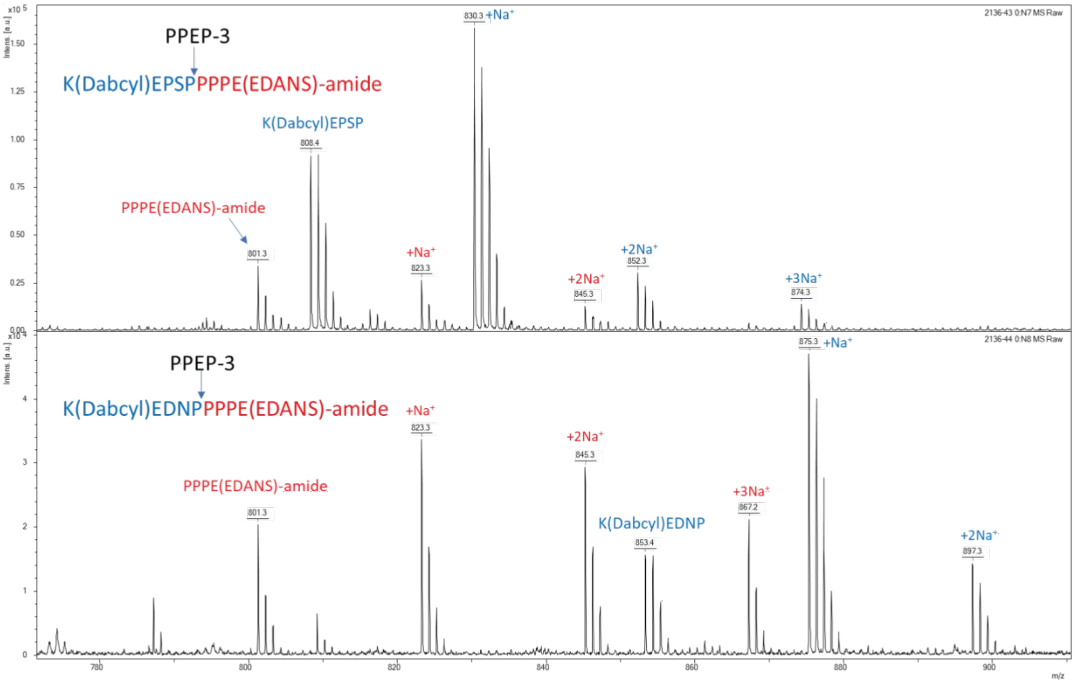

Figure S9. MALDI-ToF MS analysis of the product peptides from the incubations of PPEP-3 with the two FRET-peptides as presented in Figure 7B.

References

(1) Hensbergen, P. J.; Klychnikov, O. I.; Bakker, D.; van Winden, V. J. C.; Ras, N.; Kemp, A. C.; Cordfunke, R. A.; Dragan, I.; Deelder, A. M.; Kuijper, E. J.; Corver, J.; Drijfhout, J. W.; van Leeuwen, H. C. A Novel Secreted Metalloprotease (CD2830) from Clostridium Difficile Cleaves Specific Proline Sequences in LPXTG Cell Surface Proteins. *Molecular and Cellular Proteomics* **2014**, *13* (5), 1231–1244. https://doi.org/10.1074/mcp.M113.034728.

(2) Klychnikov, O. I.; Shamorkina, T. M.; Weeks, S. D.; van Leeuwen, H. C.; Corver, J.; Drijfhout, J. W.; van Veelen, P. A.; Sluchanko, N. N.; Strelkov, S. V.; Hensbergen, P. J. Discovery of a New Pro-Pro Endopeptidase, PPEP-2, Provides Mechanistic Insights into the Differences in Substrate Specificity within the PPEP Family. *Journal of Biological Chemistry* **2018**, *293* (28), 11154–11165. https://doi.org/10.1074/JBC.RA118.003244/ATTACHMENT/7C1197C8-11F9-407D-8FBE-BC29F2873CDC/MMC1.PDF.

(3) Hiemstra, H. S.; Duinkerken, G.; Benckhuijsen, W. E.; Amons, R.; de Vries, R. R. P.; Roep, B. O.; Drijfhout, J. W. The Identification of CD4+ T Cell Epitopes with Dedicated Synthetic Peptide Libraries. *Proc Natl Acad Sci U S A* **1997**, *94* (19), 10313. https://doi.org/10.1073/PNAS.94.19.10313.

(4) Mirdita, M.; Schütze, K.; Moriwaki, Y.; Heo, L.; Ovchinnikov, S.; Steinegger, M. ColabFold: Making Protein Folding Accessible to All. *Nature Methods 2022 19:6* **2022**, *19* (6), 679–682. https://doi.org/10.1038/s41592-022-01488-1.

(5) Hallgren, J.; Tsirigos, K. D.; Damgaard Pedersen, M.; Juan, J.; Armenteros, A.; Marcatili, P.; Nielsen, H.; Krogh, A.; Winther, O. DeepTMHMM Predicts Alpha and Beta Transmembrane Proteins Using Deep Neural Networks. *bioRxiv* **2022**, 2022.04.08.487609. https://doi.org/10.1101/2022.04.08.487609.

(6) Teufel, F.; Almagro Armenteros, J. J.; Johansen, A. R.; Gíslason, M. H.; Pihl, S. I.; Tsirigos, K. D.; Winther, O.; Brunak, S.; von Heijne, G.; Nielsen, H. SignalP 6.0 Predicts All Five Types of Signal Peptides Using Protein Language Models. *Nature Biotechnology 2022 40:7* **2022**, *40* (7), 1023–1025. https://doi.org/10.1038/s41587-021-01156-3.

(7) Madeira, F.; Pearce, M.; Tivey, A. R. N.; Basutkar, P.; Lee, J.; Edbali, O.; Madhusoodanan, N.; Kolesnikov, A.; Lopez, R. Search and Sequence Analysis Tools Services from EMBL-EBI in 2022. *Nucleic Acids Res* **2022**, *50*, gkac240–gkac240. https://doi.org/10.1093/NAR/GKAC240.

(8) Crooks, G. E.; Hon, G.; Chandonia, J. M.; Brenner, S. E. WebLogo: A Sequence Logo Generator. *Genome Res* **2004**, *14* (6), 1188–1190. https://doi.org/10.1101/GR.849004.
